# The Norrtaelje model: a unique model for integrated health and social care in Sweden

**DOI:** 10.5334/ijic.2244

**Published:** 2015-09-23

**Authors:** Monica Andersson Bäck, Johan Calltorp

**Affiliations:** Department of Social Work, University of Gothenburg, Gothenburg, Sweden; Forum för Health Policy and The Jönköping Academy of Health Improvement, Jönköping University, Jönköping, Sweden (posthumously)

**Keywords:** care coordinators, health and social care, integrated pathway, older people with complex needs, Sweden

## Abstract

Many countries organise and fund health and social care separately. The Norrtaelje model is a Swedish initiative that transformed the funding and organisation of health and social care in order to better integrate care for older people with complex needs. In Norrtaelje model, this transformation made it possible to bringing the team together, to transfer responsibility to different providers, to use care coordinators, and to develop integrated pathways and plans around transitions in and out of hospital and from nursing homes to hospital. The Norrtaelje model operates in the context of the Swedish commitment to universal coverage and public programmes based on tax-funded resources that are pooled and redistributed to citizens on the basis of need. The experience of Norrtaelje model suggests that one way to promote integration of health and social care is to start with a transformation that aligns these two sectors in terms of high level organisation and funding. This transformation then enables the changes in operations and management that can be translated into changes in care delivery. This “top-down” approach must be in-line with national priorities and policies but ultimately is successful only if the culture, resource allocation and management are changed throughout the local system.

## The intervention at a glance

### Organisation

The Norrtaelje model involves a transformation of how health and social care are funded and organised. The Stockholm County Council which is responsible for health care services and the Norrtaelje Local Authority which is responsible for social care formed a joint Governing Committee that is responsible for health and social care for the Norrtaelje population. The Governing Committee controls a public company that is responsible for purchasing and delivering that care.

### Objective

The goal of the Norrtaelje model is to promote and develop vertical and horizontal cooperation in order to better respond to the common needs for integrated health care and social services. The model is characterised by (1) funding responsibilities for a single population, (2) increased focus on health promotion for the population, and (3) a common and integrated health and social care organisation to achieve greater patient and user benefit.

### Target population

The Norrtaelje model is responsible for care of the entire population of the region. Individuals aged 65 years and older are one of the main groups cared for and within the model specific target populations such as older people who are to be discharged from acute care and older people in the community who need home care services.

### Approach

The model supported the development of a range of different care paths for older people and the restructuring and redeployment of providers. There was an emphasis on the management team, using care coordinators and on developing pathways and plans around transitions in and out of hospital from nursing homes to hospital.

### Timeline

For the first two years (2004–2005) the focus was on high level planning around the transformation. The fundamental changes in organisation and funding took place in 2006. The changes in the care delivery were observed from 2006 to 2012.

### Results in brief

It took time for the model to develop from planning and organisation clinical implementation. The transformation occurred within the existing budget and did not involve new funds. With the implementation of the model, the Norrtaelje region improved its rank in some key performance measures compared to other regions in Sweden.

## Introduction

The Norrtaelje model involved the transformation of the financing, funding and management of health and social services. The Norrtaelje model created an integrated provider and purchasing system for health and social care. In the rest of Sweden, county councils fund and control health care and municipalities fund and control social care services. In the Norrtaelje model, the Stockholm County Council and the Norrtaelje Local Authority created a joint political governing committee. This joint committee steers a public company – Tiohundra AB – and that company manages a comprehensive set of health and social care services. The partners also created an integrated administrative structure that executes the policy and mission of the shared effort and that collects payments from different sources and reimburses providers.

The goal of the Norrtaelje model is to promote and develop vertical and horizontal cooperation in order to better respond to the common needs for integrated health care and social services. The model is characterised by (1) funding responsibilities for a single population, (2) increased focus on health promotion for the population, and (3) a common and integrated health and social care organisation to achieve greater patient and user benefit. The model has a particular focus on the group of older adults with complex needs. The aim is that older people should be able to remain in their own homes as long as possible, and that older people should not suffer from poor integration among providers from different organisations, management systems, cultures, traditions and procedures.

The Norrtaelje model addresses a problem faced in many countries – providing integrated health and social care in a decentralised system where these two facets of care are funded and controlled by different levels of government. This integration is particularly important for older people with complex needs. Sweden like other countries is facing the demographic challenge of a growing population of older people. In 2011, 1.8 million Swedes were aged 65 or older, representing nearly 19% of the total population of 9.5 million inhabitants. By 2020, the share will approach 25% [1] and the number of 80- to 99 year-olds is expected to be more than double from 2020 to 2060 [2, p. 27]. This will put enormous pressure on the financial sustainability of the Swedish system. Along with the financial pressure there are concerns about the quality of care. Older Swedes experience lack of security, poor continuity and coordination of actions from different health and social care providers [3–5]. Along with the national context, there were local circumstances that helped propel the development of the Norrtaelje model. Financial concerns led to a threat of closing down the local Norrtaelje Hospital, something that the employees as well as the local residents did not want to happen The Norrtaelje model was the result of innovative and collective efforts to find a better way of working together in order to meet the desire of Swedes’ for a simpler more effective way to navigate in health and social care [6].

**Figure 1.**
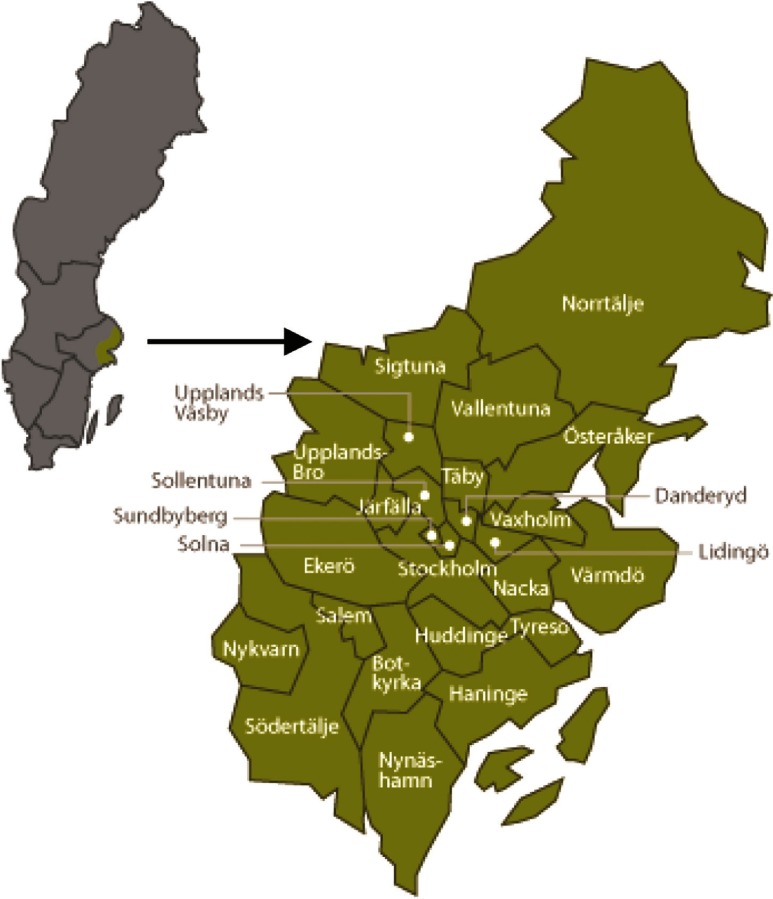
The location of Norrtaelje within Stockholm County Council in Sweden.

Norrtaelje is a local authority area situated in the north of Stockholm region (Figure 1). In 2012, the municipality had a population of about 56,000 people, more than twice as many as those who lived there in the 1970s. The municipality is a summer tourist destination and the summertime population is approximately 120,000 people. Only 31% of the inhabitants live in the main city and the region covers 2114 km^2^ land and sea, including 63 small villages, numerous lakes and an archipelago with 10,000 islands of which seven are populated. Prior to the Norrtaelje model, the specialist hospital and the primary health care in Norrtaelje were managed, owned and tax-funded by the Stockholm County Council. This meant that all physicians and health care personnel were salaried employees with the exception of two independent medical family practice contractors. The central purchasing unit in Stockholm County Council had 40 different contract agreements with the hospital and with primary health care in the area, as well as with specialised health care outside of the area (totaling 200 M SEK or 20 M Euro for the Norrtaelje population). Norrtaelje health care and primary care were thus governed by the Stockholm's central political body of elected politicians. The local Norrtaelje Local Authority provided, owned and funded service and long-term care for older people, the physically disabled and those suffering from long-term mental illnesses (total budget, about 700 M SEK or 70 M ECU). The municipality operated their own public nursing homes and home care services. In addition, the municipality provided social services financial assistance, childcare, school health services and environmental health, as well as a number of other local non-health services.

The new Norrtaelje model brought the Stockholm County Council together with the Norrtaelje Local Authority in a new structure that is summarised below. The shared approach to policy and financing created a climate that allowed better integration of resources and staffing and that provided an impetus for developing the information technology and documentation strategies that enable integrated care. The Norrtaelje model created an organisational environment that allowed providers of health and social care services to work together to provide the right mix of services, including preventative services and services that focused on clients making transitions across sectors.

**Table:**
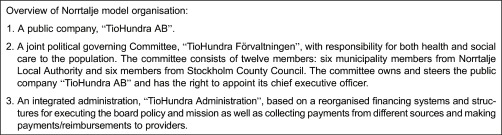

## Target population

The Norrtaelje model was designed to cover the entire population of the region. However, the programme developed a specific set of programmes for older people in the region around the following objectives:

- To get their health and social problems addressed/solved, and also to satisfy wishes related to health, social care and continuation of a good life in general despite age, chronic conditions and multiple morbidities;
- To experience a higher degree of security in their daily life, continuity and coordinated/comprehensive help from health and social professionals, carers and organisations;
- No older person should be a victim of poor integration, be outside of everyone's responsibility (In Swedish the idiomatic expression is referred “Nobody should fall between two chairs”. The symbol for Tiohundra AB is a showing two chairs attached to each other.);
- Specific objectives consider good palliative care;
- Prioritised prevention work;
- Good care for people with dementia;
- Good and safe medication.

The increasing number of older people found in Sweden is particularly apparent in Norrtaelje, where the proportion of 65 and older, and the proportion of people aged 80 and older have increased more than in the rest of Stockholm County and in the average of Sweden. In 2005, 19.6% of the population were 65 and older, and 5.7% of the population were 80 years and older in Norrtaelje – in 2010 these figures had risen to 23.1% for those aged 65 years and older and 6.0% for those who were 80 years and older.

As outlined in the approach to care, although the Norrtaelje model was designed to provide care to the entire population of the region, the implementation of the model involved developing specific approaches to care that were targeted on well-defined groups of older adults.

## Description of the care practice

The overall approach is based on joint health, care and medical documentation, health care planning, activities for rehabilitation, prevention and safe medication, home service, home care and primary care. It involved the creation of teams for specific purposes and the creation of fora for meeting, improvement and training. The approach built on the overall approach to care improvement used in Sweden that involves defining and implementing pathways and processes for defined populations or interventions.

Key to the approach was a decision that all providers, public and private, who want to serve the population in the area must act within an integrated chain of home care services, health care and rehabilitation. The overall aim for the Norrtaelje model and the public company Tiohundra AB was better coordination between health and social care organisations and focused vertical and horizontal integration built on overall health care planning, information exchange and common problem solving (e.g. management groups) and interactions between professionals and teams, making it possible to better coordinate economic and human resources. Thus, activities are taking place at different hierarchical levels simultaneously.

The approach had a focus on improving processes of care that were relevant for older people with complex needs**. Two areas provide examples of the how the combination of improved pathways, new roles and teams are applied in the model – the provision of health, social and rehabilitation care for older people at home or in residential care and planning for older people when they are discharged from hospital.

For patients at home/in nursing homes, a process was created for the integrated provision of health, social care and rehabilitation (IVOP). This process considered home service/residential care and their connections with district nurses, physicians and paramedical staff at home care, primary care, rehabilitation as well as at the hospital. The purpose of the IVOP was to prevent visits at emergency departments and hospitalisation for this fragile group of older people with multiple problems. This population was at high risk of repeated emergency department visits and hospitalisation. The model allowed better coordination of services than elsewhere in Sweden where health and social care are organised and funded at different levels. In Norrtaelje, home care is organised together with home-help service and transferred from primary care, while in other parts of Sweden it is still a county-based care unit. Under the new model, the district nurses were able to meet regularly with home care workers and the health care assistants/enrolled nurses. The home care staff are able to identify individuals with increasing needs and communicate that to the district nurse who can provide extra services. The changes in organisation and responsibility allowed for the identification of a care coordinator for each older person. This person supports the older adult with complex needs to navigate between homecare, rehabilitation and hospital/primary care. He/she is most often a home care worker, and contributes to secure well-coordinated interventions from each of the different professionals/specialties by having information and ability to influence, coordinate all interventions around a person with complex needs.

The care coordinating role is also undertaken by the so-called “health care coaches” who can be a district nurse or a nurse with speciality training. These health care coaches are on those older people who are frequently hospitalised.

A care manager (biståndshandläggare) a formal role within the Swedish overall system, has the delegated right to independently make decisions about help, service and assistance for older people in need of home care service and residential nursing home care. This decision is adapted to meet the patient's particular needs and wishes [7]. The decisions are made in line with the national Social Service Act of 1982 and recognise that individual has the right to a dignified life built on integrity, self-determination and care of good quality [8]. Recently, private providers are available. If the client chooses a private provider, the needs of services/help/care defined by the care manager is still public funded, but the client can buy extra services at a reduced price [9]. Like many municipalities Norrtaelje has drafted guidelines providing rather detailed rules for decision-making [10].

For older people who are hospitalised, care planning is undertaken by the physician in charge and the responsible case manager, a so-called patient responsible nurse. In the case of an older adult “enough medically treated to be discharged from specialist care”, a care plan is made for the future care outside the hospital. The case manager at the hospital then calls for a meeting together with a care manager and other involved professionals, in order to make a plan care that will help ensure a good transition from the hospital to appropriate living and care at home/in a residential home. This role is particularly important when it is felt that at discharge the older person will require home care or will be discharged to a nursing home. As part of this effort, representatives from the emergency department and the wards created and implemented routines and checklists to improve the processes. This process included making a care plan available for each patient leaving the hospital. The plan summarises the hospital visit and indicates how care will proceed at home.

The care activities are planned together with the patients and his/her relative/s. The planning involves personnel from relevant departments, who possess the necessary skills to meet the patient's needs after discharge from the hospital. Two persons were appointed and responsible for care planning in each ward at the hospital, and they in their turn started to build up news ways of working in their wards. Along with this, a team or an individual was made responsible for implementing follow-up care plans [11, pp. 75–76].

Other attempts were designed to improve the information and communication processes between the hospital and the primary care centre as well as between primary care and home care. Concerns over the lack of overview, communication and knowledge exchange between providers in different organisations were identified [11, p. 44]. In response, meetings with managers and professional in the different organisations took place in order to design particular routines and practice for information documentation in the two information systems to ensure a good information flow and continuity of care between different providers. To quote a study of the model “so the first thing we did was to have monthly meetings where all activity managers were asked to present their own part of the activity and what they were doing, their competences, their ambitions and possibilities. We really tried to merge the different approaches, and bridge the gap between old job types” [12, p. 116]. Sweden has a long track record in developing care plans for specific conditions such as hip fracture and stroke and these were reviewed and monitored.

The model supported the development of a range of different care paths for older people and the restructuring and redeployment of providers. In the strategy for caring for older people with complex needs, there was an emphasis on using case managers and on developing pathways and plans around transitions in and out of hospital from nursing homes to hospital.

## Implementation

The implementation of the model occurred over the period from 2006 to 2012 and can be divided into four phases. The first two phases (2004–2006) involved the transformation of the funding and organisation of health and social care. It was not until 2007 that changes occurred at the level of clinical care to populations. In 2010, there were fundamental changes in Swedish law that had impacts on how services were offered and purchased. The four phases are summarised below and the first three phases are described in more detail by Øvretveit et al.[13]

### Phase 1: planning/preparation (2004–2005)

Phase 1: planning/preparation (2004–2005) plans were created and agreed by the health and community committees, and by Stockholm county council. The decision was to save the hospital, given that the organisation streamlined it operations and made savings of 20 M SEK (2.1 M Euro) for year 2004. Three major public meetings took place open for the inhabitants and other interested, where plans were presented, and views and opinions were exchanged among decision-makers and the public regarding the proposition for a new system for integrated health and social care. Negotiations were held with 15 different trade unions to conclude agreements about the transfer of employment contracts and working conditions for all involved staff. Information was spread to the staff through newsletters, meetings, publicity and website. Discussions took place for planning a new patient care information system and a medical record system to be shared between providers.

### Phase 2: macro-structure establishment (2006)

Administrative system and routines were developed for activities and financing embracing internal needs and requirements, to harmonise both county and municipality demands. The health and social care were commissioned through the governing Committee covering tasks for revenue collection and fund pooling. In 2006, the political board and the administration were split from the public company in order to assume the different roles as purchaser and provider. Overall, directions and policies are given by the Stockholm County, to which the Norrtaelje model belongs. A new system for incentives and renewed financial system for reimbursement was adopted. Employment contracts were transferred to the new organisation. The actual formation of the new management structure took place in 2006. Some people changed employment positions and new management committees and reporting arrangements were introduced. The recruited CEO entered the post on the 1st of January 2006.

### Phase 3: structure for micro-integration (2007–2009)

Planning of new financing and organisation of the patients/clients in three major groups defined according to age 0–18, 18–64, above 65. (65 is the official age for getting a retirement pension in Sweden, although professional variations exist.) Contracts were set up for the three different patient groups and the data required to be reported to purchaser by the provider organisation TioHundra AB. From then on, planning took place of new reporting system of finance, information and human resource in accordance with the old auditing requirements of the rest of Stockholm County and the municipality goal-reports. The hospital launched a new financial system in accordance with the requirement from the three new care groups.

Other actions for micro-integrations concerned creating a new and coordinated patient pathway for stroke patients’ entire care episode, as well as creating new organisation for an integrated care of elderly and home care service. Actions to spread and share the “The vision and goals of TioHundra” were undertaken among all managers. Discussions about efficient processes took place in order to meet the goals and needs and how to measure compliance, the effects and perceptions. Actions were furthermore implemented towards a coordinated development of health and care.

### Phase 4: integration and competition (2009–2012)

In 2010, the Swedish Act on System of Choice in the Public Sector implemented it in the area. It made it possible for the citizens to select their provider of primary care as well as provider of home-help service or nursing homes if needed. These actions implied increased competition for the company TioHundra AB, and a situation with eight different primary care and elderly care providers within the area of Norrtaelje, instead of one major complemented by some private family doctors. This made the integration even more difficult as several new actors entered the scene and no natural interaction points were identified.

## Evaluation of impact

Three separate and independent evaluations of the Norrtaelje model were conducted [14–16].

**Figure 2.**
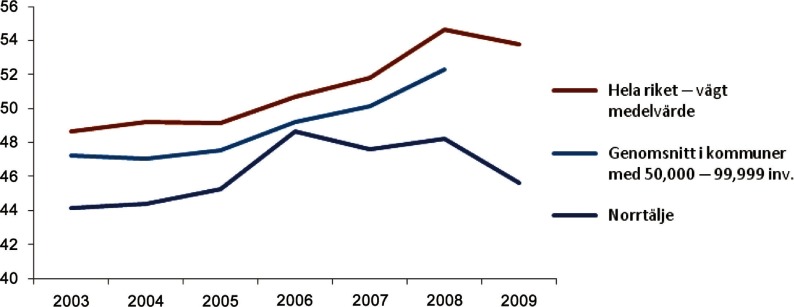
Development of total costs for elder care, 1000 SEK per inhabitant 65+ years.

The first, the MMC evaluation used data from the National Board of Health and Local Authorities and Regions project “Open comparisons” to compare costs of different municipalities [17]. The evaluation revealed that between 2005 and 2009, Norrtälje climbed from 171st place nationally to 61st place in terms of the difference between net costs and standard costs of care. Focusing on gross costs for people aged 65+, getting home-help service Norrtalje had moved from 206th place nationally in 2001 to 29th place in 2011. [17, May 2013] As shown in Figure 2, the total costs for elder care in Norrtalje peaked in 2006 and then were stable or decreased and remained lower than the Swedish average and compared to municipalities of equal size.[14, p. 45] The red top line symbolise the average development of total costs for the entire Sweden, the middle light blue corresponds to the average development of total costs in municipalities with 50,000–99,999 inhabitants, and the dark blue bottom line represents the value in Norrtaelje.

**Figure 3.**
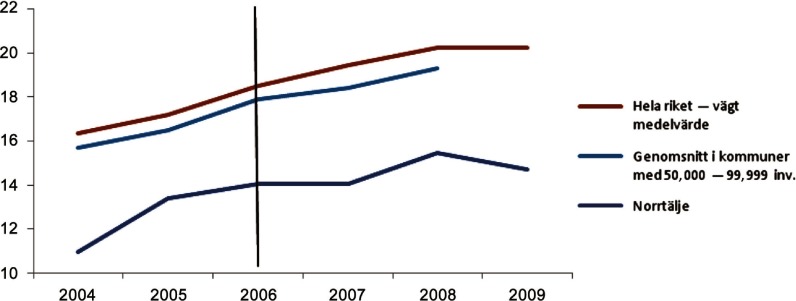
Total costs for ordinary living with help from home help service, 1000 SEK per inhabitant 65+ years.

As Figure 3 shows, when only the cost for home help service to older people at home the cost trend was similar to the national average, but the level of costs was consistently about 50% less than the Swedish average.[14, p. 46]

Over this period, the number of appointments with primary care physicians and nurse per capita.[14, pp. 47–48]

The second evaluation examined the impacts on coordination between health and social services. Their focus was on tools for integration of purchasing, such as specification of mission, models for resources allocation and follow-up, as well as tools for integration of provision (e.g. infrastructure for planning, implementing and following-up activities within an organisation). They found that traditional boundaries were removed and that new integrated review procedures were developed.[15, p. 5]

As a consequence of a new organisation and integrated functions, they found that resources had been pooled and redistributed. Their report stressed the importance of an advanced purchaser organisation that is able to support integration regarding all activities: purchasing, agreements, provision, follow-up and control, in particular as the number of providers and the competition increased [15].

The third evaluation showed that the geriatric care had become a focus and that there was better access for older people with complex needs [16]. The found more older adults living in nursing homes now had their own geriatrician, which meant a higher degree of competence and continuity of care [16]. They concluded that as a result of the integration of activities, information and communication had improved among different groups of professionals. Based on interviews, they concluded that there was an increased willingness for collaboration and joint problem-solving.[16, pp. 7–8] Based on these evaluations,[14–16] SALAR, the legal commissioner, concluded that the Norrtaelje model had resulted in a substantial restructuring of care for elderly, that wait lists for nursing homes had decreased, that the share of elder adults living in nursing homes was decreased, that costs decreased and that quality had improved [18].

## Barriers and facilitators

At the broad contextual and systemic level, there was a range of barriers and facilitators to the fundamental transformation of how health and social are organised that is the core of the Norrtajle model. The historic and well-entrenched gaps between health and social care were more profound and difficult to bridge than had been thought. There were important differences in cultures, traditions, professional, legislations, ways of working and communicating. The model required basic and fundamental changes in how individuals were paid and organised, for example, it was necessary to get a system in place for distribution of salaries in the newly constructed organisation to 3000 employees, previously employed within two different structures either the municipality or the county.

Existing and changing national legislation also had important impacts on the implementation of the integrated care model. National laws prevented “municipality” personnel from having direct access to health care client records, and vice versa for “county” staff having access to documentation for social service. The implementation of the Act on System of Choice in the Public Sector has been and is a huge challenge to the Norrtaelje model. Its focus on competition and multiple service providers has had negative effects on integration and collaboration between health and social care. However, on the other side of the ledger, changes in regulations around that enabled shared boards made the model possible in the first place.

Perhaps the biggest local contextual factor was the threat to close the local Norrtaelje Hospital. There was strong local opinion against such a change, among both inhabitants and the staff of the hospital. This external threat was the impetus for a spirit of shared commitment and collaboration. Norrtaelje is a relatively small community. In this context, health and social care professionals are aware of and know each other well and many have worked together. Not only that they know the older inhabitants and especially those with multiple problems are can see how the changes can help those people. Ultimately, as may occur in many relatively small and isolated regions, there is a local spirit that allows for and drives change – in this case it was known as the *Norrtaelje spirit* [14].

From the outset, there was a concerted effort to bring people together to define a common vision, to share goals and activities among all managers. The CEO's commitment to engage all managers in the integration work, considering the company as one actor and not as had been the case previously – different actors within different sectors. Initially, the management team consisted of all, 15 senior managers responsible for a division or for a support function. It was a good base to build the strength of leadership. This meant that the model had continuity and support of senior management. To quote from a statement by the Development Strategist (Oct 2012) *“To have a big management team*, in the beginning, was very good I think. It meant that all managers did take part of each other's concerns and became aware of the on-going activities in all the divisions and other parts of the organisation. Accordingly, it meant a flatter organisation. I think this allowed managers to get a deeper understanding of each other's activities and that all the managers worked on the process of linking different activities together.”

Concrete integration activities were undertaken with help from consultants, within an overarching organisational development work based on different kinds of care processes – regarding different diagnosis group and generally linking the patient's/client's pathway through hospital, primary, home health care and rehabilitation with care and services in the patient's home and a nursing home. However, the staff showed a certain degree of project fatigue due to the number of simultaneously project. The pace and amount of reorganisation and major project start-up seemed to take a lot energy from the active staff [19, p. 7].

Initially, there were problems due to the large number of information technology systems in place that were not designed to share information or support integrated care. A process of integrating the existing information technology system subsequently took place. By 2013, there are only three major information technology systems instead of 14 used when the model was established. Decisions were taken and actions were made to unify different actors around a joint health and medical documentation system for patients in their charge allowing spread of information and communication exchange between actors for hospital, medical service and care, i.e. they improved the information technology systems towards integrated processes. The decision was taken that Norrtaelje, as one among several health care areas, should connect to the National Patient Overview (Nationell patientöversikt) [11, p. 61].

Although there was a commitment to common funding for health and social care it was difficult to implement. Despite the fact that funds for the Norrtaelje model were provided by both the Stockholm County Council and Norrtaelje Local authority, no deep pooling of resources was made in order to materialise an integrated commissioning for patient groups, such as older adults with complex needs. An international group of health care specialist strongly advised commission on an integrated basis in order to strengthen and fully integrated care, instead of payment for a series of individual activities. One recommendation was to design a pilot of totally integrated commission for the patients 75 years and older based on population data on cost of all care, excluded elective care [19, p. 8]. However, instead of supporting the Norrtaelje model, the political committee put an even higher emphasis on separate reimbursements and detailed agreements based on the traditional health and social care structure.

Successful multi-professional teamwork was found around the older adults from home care workers and nurses/district nurses with support from the chief physician home care for older people living at home, and with additional support from the geriatricians in the nursing homes. The district nurses and the physicians had important coordination roles in areas such coordinating regular care with specialist care. However, the hospital was to some extent isolated from the rest of the care chains. Symon et al. however concluded “interestingly, although we acknowledge that a lot of efforts are being put into communicating this common vision, staff at different levels did not necessary feel that others shared their understating of their objectives. In particular, the hospital was often mentioned as sitting in slight isolation to the rest of the organisation” [19, p. 3]. Furthermore, it could be concluded that several functions were used to coordinate the individual patient's/client's pathway at different levels such as formal the care managers, the responsible case managers, the patient responsible nurses, the district nurses and newly defined roles in Norrtaelje, such as the chief physician home care, care coordinators and “health care coaches”. Each coordinating role springs from a specific organisation and it is still unclear how it improve the overall view of the patient/client.

In Norrtaelje it was possible to actively engage both the community and the workforce in designing and making the change. The challenge of changing organisational culture – a prerequisite for integrating care – may be more difficult in other settings that are either larger or that are in more open environments. Although the success of the Norrtaelje model is based in part on its local focus, it is clear that these local efforts can be affected both positively and negatively by the broader environment. The Norrtaelje model was able to develop only because the Stockholm City Council was willing to be a partner but as the policies of that level of governance change and as other national initiatives, such as the patient choice legislation come into play, the local programme had to adapt in order to survive. This is a typical example of how conflicting policy goals at both national and local level interplay and are interrelated.

## Conclusion

A key challenge was the underestimation at the outset of the complex and fundamental nature of the change in organisation and funding. Making a change like Norrtaelje model requires a systematic, strategic plan and involvement from external expertise [20]. Moreover, it can take time for the broad changes at the macro-organisation level to result in the micro-level changes in care provide. Complex structural and contextual aspects hindering the integration, such as legal aspects for documentation systems and different traditions, legislation, professional training, status and working practice remained long after the implementation started. National changes in favour of competition and multiple actors made it even more difficult for integration as new actors are entering at the same time as established actors try to find ways to integrate. Several facilitating factors did contribute to the success of the model including the threat to close the local hospital which helped engage the community, the local Norrtaelje spirit, the intense management work, commitment, shared vision, a common management team, and training and concrete integration activities. The integration activities included mapping processes and defining critical moments and need for common efforts as well as transfer tasks from one professional to another or from one organisation to another. In the end, although the process was slow and is still evolving, the Norrtaelje model was able to change how care was delivered, to improve the quality of care and it did this with no extra funding. It was made possible by a shared vision and commitment to make that vision a reality.
